# Comprehensive cell surface protein profiling of human mesenchymal stromal cells from peritoneal dialysis effluent and comparison with those from human bone marrow and adipose tissue

**DOI:** 10.1007/s13577-023-00971-x

**Published:** 2023-08-21

**Authors:** Ganggang Shi, Chong Yang, Lan Zhou, Ming Zong, Qiunong Guan, Gerald da Roza, Hao Wang, Hualin Qi, Caigan Du

**Affiliations:** 1 Department of Colorectal Surgery, The Second Hospital of Tianjin Medical University, Tianjin, People’s Republic of China; 2 Department of Urologic Sciences, The University of British Columbia, Vancouver, BC Canada; 3 Organ Transplantation Center, School of Medicine, Sichuan Academy of Medical Sciences, Sichuan Provincial People’s Hospital, University of Electronic Science and Technology of China, Chengdu, Sichuan People’s Republic of China; 4 Department of Urology, Shanghai United Family Hospital, Shanghai, People’s Republic of China; 5 Shanghai East Hospital, Tongji University School of Medicine, Shanghai, People’s Republic of China; 6 Division of Nephrology, Department of Medicine, The University of British Columbia, Vancouver, BC Canada; 7 Department of General Surgery, Tianjin Medical University General Hospital, Tianjin, People’s Republic of China; 8 Department of Nephrology, Shanghai Pudong New Area People’s Hospital, 490 Chuanhuan Nan Lu, Pudong New Area, Shanghai, 201299 People’s Republic of China; 9 Jack Bell Research Centre, 2660 Oak Street, Vancouver, BC V6H 3Z6 Canada

**Keywords:** Peritoneal mesenchymal stromal cells, Cell surface protein phenotype, MSC lineage, Bone marrow MSCs, Adipose MSCs

## Abstract

Peritoneal mesenchymal stromal cells (pMSCs) are isolated from peritoneal dialysis (PD) effluent, and treatment with the pMSCs reduces peritoneal membrane injury in rat model of PD. This study was designed to verify the identity of the pMSCs. pMSCs were grown in plastic dishes for 4–7 passages, and their cell surface phenotype was examined by staining with a panel of 242 antibodies. The positive stain of each target protein was determined by an increase in fluorescence intensity as compared with isotype controls in flow cytometrical analysis. Here, we showed that pMSCs predominantly expressed CD9, CD26, CD29, CD42a, CD44, CD46, CD47, CD49b, CD49c, CD49e, CD54, CD55, CD57, CD59, CD63, CD71, CD73, CD81, CD90, CD98, CD147, CD151, CD200, CD201, β2-micoglobulin, epithelial growth factor receptor, human leukocyte antigen (HLA) class 1, and, to a lesser extent, CD31, CD45RO, CD49a, CD49f, CD50, CD58, CD61, CD105, CD164, and CD166. These cells lacked expression of most hematopoietic markers such as CD11b, CD14, CD19, CD34, CD40, CD80, CD79, CD86, and HLA-DR. There was 38.55% difference in the expression of 83 surface proteins between bone marrow (BM)-derived MSCs and pMSCs, and 14.1% in the expression of 242 proteins between adipose tissue (AT)-derived MSCs and pMSCs. The BM-MSCs but not both AT-MSCs and pMSCs express cytokine receptors (IFNγR, TNFI/IIR, IL-1R, IL-4R, IL-6R, and IL-7R). In conclusion, pMSCs exhibited a typical cell surface phenotype of MSCs, which was not the same as on BM-MSCs or AT-MSCs, suggesting that the pMSCs may represent a different MSC lineage from peritoneal cavity.

**Supplementary Information:**

The online version contains supplementary material available at 10.1007/s13577-023-00971-x.

## Introduction

There has been a consensus definition of human mesenchymal stromal cells (MSCs) from the International Society for Cellular Therapy, the minimal criteria for defining MSCs, as follows: (1) MSCs are the fibroblast-like adherent cells growing on untreated plastic culture plates, (2) MSCs display a positive expression of CD105, CD73 and CD90, and a negative expression of popular hematopoietic marker CD45, CD34, CD14 or CD11b, CD79 or CD19 and human leukocyte antigen (HLA)-DR surface molecules, and (3) MSCs have multipotentiality to differentiate into adipocytes, osteoblasts and chondrocytes under in vitro culture conditions [1]. As of today, different MSC populations or lineages have been isolated from a variety of human organs or tissues including the bone marrow (BM), adipose tissue (AT), birth-derived tissues such as umbilical cord (UC), dental pulp and endometrium for development of new therapies based on their capabilities of self-renewal, immunomodulation, and multidifferentiation potential or tissue regeneration or repair in vivo [2–4].

As of year 2020, there were 1,426 clinical trials registered in the U.S. National Library of Medicine (https://clinicaltrials.gov) to test the safety and efficacy of autologous or allogeneic MSCs for treatment of a variety of diseases occurring in all different organ systems [5], and among them over 300 clinical trials have been completed [6]. Except potential risk of promotion of tumor growth and metastasis by MSCs [7] and version loss in 3 patients by direct injection of AT-MSCs into the eyes [8], all published studies have confirmed the safety of MSC-based therapies without notable adverse effects [6, 9, 10], but the efficacies have fallen short of expectations—either neutral or marginally significant [6, 9], which may be due to several challengers in this field including immunoincompatibility between donors (MSCs) and recipients, and heterogeneity in functional cell surface markers, immunomodulation and differentiation potential that have been extensively discussed in recent literature [6, 10–14]. Furthermore, it has been known that adult MSCs naturally reside in specialized tissue structures—stem cell niches that provide the microenvironment and extracellular signals from growth factors and cell-to-cell interactions for maintaining MSC stemness and differentiation potential [15], and there is different epigenetic memory between BM-MSCs and AT-MSCs that regulates differentiation to the osteoblastic or adipocytic lineage [16], implying that the efficacy of MSCs may depend on their sources and target tissues. Recently, Hoang DM et al. [10] have proposed that MSC origin may play a key role in the downstream application—therapeutic use. Therefore, any types of MSCs before clinical testing may require comprehensive characterization of its phenotype, trophic factor expression and possible action mechanisms in order to development of a unique therapy based on each type of MSCs.

Adherent cells were isolated from otherwise discarded peritoneal dialysis (PD) effluent from PD patients by growing in plastic culture dishes and were first identified as MSCs based on the MSC minimal criteria including trilineage differentiation into adipocytes, osteoblasts and chondrocytes [8]. These MSCs express some classical MSC marker CD29, CD44, CD73, CD90 and CD166 and are negative in the expression of CD14, CD34, CD79a, CD105, CD271, HLA-DR, STRO-1 and SSEA-4, termed as PD effluent-derived MSCs (pMSCs) [17–20]. More interestingly, the therapeutic effect of pMSCs is stronger than that of UC-MSCs on the protection of peritoneal membrane (PM) from PD solution-induced injury in a rat model of PD [20]. In addition, when autologous pMSCs are used specifically for treatment of PD-induced PM injury in the same patients, not only there are no immunoincompatibility issues between injected cells and patients, but also because of epigenetic memory pMSCs may be superior over any other types of MSCs in the homing, differentiation and other biological activities in the peritoneal cavity. Therefore, we propose that the autologous pMSCs from PD patients may become a promising candidate for a MSCs therapy in the treatment of PM dysfunction in the same PD patients. The objective of this study was designed to characterize the cell surface phenotype of the pMSCs using a comprehensive panel of antibodies against 242 cell surface proteins.

## Materials and methods

### Ethics approval

The study was conducted in accordance with the Declaration of Helsinki, and approved by the Office of Research Ethics/Clinical Research Ethics Board of the University of British Columbia and Fraser Health Authority (British Columbia, Canada) (protocol number: H15-02466).

### Sampling

PD effluents are considered waste products from PD patients and are discarded at the end of PD. With the approval protocol (H15-02466) the research laboratory requested PD effluent collection each time, followed by approval from the PD clinic, so that any excessive sample collection could be avoided. The patient’s information (except ethnicity, gender, age, and PD solution used) on the PD effluent bag was anonymized by the PD clinic before delivery to the laboratory to protect patient’s privacy. There was no ethical concern to any patients.

As listed in S1 Table, PD effluents (*n* = 6) were collected from PD patients who received Dianeal or Physioneal PD solution-based PD therapy within 4 weeks in Royal Columbia Hospital (New Westminster, BC, Canada). Because the PD effluent collection did not have any impact on patient care as mentioned above, after dialysis patients gave verbal informed consent by a nurse in the PD clinic for the use of otherwise discarded PD effluent for research purpose, which was witnessed by patient’s family member in accordance with the protocol H15-02466. Patient information on the PD effluent bag was de-identified by the same nurse prior to handing it over to the research laboratory. The authors in this study had no access to the information that could identify individual participants during or after sample collection.

### Isolation and growth of pMSCs

The cellular component including pMSCs was isolated from the PD effluents simply by pelleting using the centrifugation, and pMSCs were isolated and expanded by growing in Dulbecco’s modified Eagle’s medium/Ham’s nutrient mixture F12 (DMEM/F12) containing 10% fetal bovine serum (FBS) (Thermo Fisher Scientific, Waltham, MA, USA) in plastic culture dishes as described previously [17, 19].

### Determination of cell surface protein expression using a flow cytometry

After growth and expansion of 4 to 7 passages in plastic culture dishes, pMSCs were detached with 0.05% trypsin–EDTA solution (Sigma-Aldrich Canada, Oakville, ON, Canada), followed by washing with cold phosphate buffered saline (PBS). Finally, cells were suspended in EDTA-containing BD Pharmingen Stain buffer (EPS) for staining of cell surface antigens using a Human Cell Surface Marker Screening Panel of monoclonal antibodies (BD Lyoplate™, BD Biosciences, Franklin Lakes, NJ, USA) following manufacturer’s instruction. This panel consisted of 242 primary antibodies to surface proteins (S2 Table), and a complete set of both mouse and rat isotype control antibodies for background staining was included. AlexaFluor® 647 conjugated goat anti-mouse or rat Ig were used as secondary antibodies.

In brief, a single-cell suspension of pMSCs in EPS (5 × 10^4^ cells per well) was incubated with the primary antibody in 96-well plates. Three experimental groups were included: (1) blank or buffer control, in which no primary antibody was added, (2) isotype antibody, an isotype control antibody was used as the primary antibody, and (3) cell surface marker, a monoclonal antibody to a cell surface protein was used as the primary antibody. After incubation in the dark for 30 min at 4 °C, pMSCs were washed with EBS, followed by staining with the secondary antibody (1:200 dilution) in the dark for 30 min at 4 °C. Finally, the cells were washed twice with EPS. The mean fluorescence intensity (MFI) of each sample measurement was determined using a calibur flow cytometer (BD Biosciences). Data were analyzed with FlowJo software (FlowJo, LLC., Ashland, OR, USA).

The raw MFI values of all three groups (blank control, isotype antibody control, and cell surface marker) were initially collected based on the flow cytometry histogram. The MFI of specific staining with the isotype antibody or the antibody to cell surface markers was counted by subtraction of background staining (blank control).

### Statistical analysis

Data were presented as the mean ± standard deviation (SD) of samples from six patients. The differences of the MFI between isotype antibody control and the antibody to each cell surface protein were compared by using *t*-tests (two-tailed distribution) of Prism GraphPad software version 4 (GraphPad Software, Inc., La Jolla, CA, USA) as appropriate. A *p* value of ≤ 0.05 was considered significant.

## Results

### Cell surface protein phenotype of pMSCs in cultures

The mean fluorescence intensity (MFI) of staining of 242 cell surface protein markers on pMSCs from six patients was presented in S2 Table. As compared with the variation of MFI of the staining with isotype antibody controls (140.41 ± 782.85, *n* = 22), the expression of these 242 cell surface proteins could be classified into four groups based on the difference of MFI (*p* value of statistical analysis) between target protein staining and isotype control as follows: (1) A high level group, in which the MFI of identifying marker was high in every patient sample with *p* < 0.001 (Table 1); (2) a low to moderate level group, in which the marker expression was detected at a low to moderate level in every patient sample with *p* < 0.05 (Table 1); (3) a heterogeneous group, in which the expression of the marker in one to three out of six patient samples was negative but with *p* < 0.05 as compared with those of isotype antibody staining (Table 1), and (4) a negative group, representing some common MSC markers but found negative on pMSC with *p* > 0.05 (Table 2).

**Table 1:** Different levels of cell surface protein expression on peritoneal mesenchymal stromal cells (pMSCs)

Protein	Functions (gnecards.org)	MFI (mean ± SD)	*p* value*
	High level group		
CD9	A member of the tetraspanin family, plays a critical role in the suppression of cancer cell motility and metastasis	2855 ± 1873	1.03 × 10^–5^
CD26	Dipeptidyl peptidase 4, highly involved in glucose and insulin metabolism, and in immune regulation	3563 ± 1566	5.04 × 10^–8^
CD29	Integrin subunit β1, involved in cell adhesion	2354 ± 1794	1.00 × 10^–4^
CD42a	Glycoprotein IX platelet, functions as a receptor for von Willebrand factor	2039 ± 1930	9.05 × 10^–4^
CD44	A cell-surface receptor for hyaluronic acid and other ligands (i.e. osteopontin, collagens, and matrix metalloproteinases), involved in cell–cell interaction, cell adhesion and migration	29,839 ± 13,192	2.48 × 10^–11^
CD46	A type 1 membrane protein, a regulatory part of the complement system for inactivation of C3b and C4b	9918 ± 3319	5.53 × 10^–13^
CD47	An adhesion receptor for thrombospondin 1 on platelets, and in the modulation of integrins, has a role in cell adhesion	2297 ± 881	3.79 × 10^–6^
CD49b	Integrin subunit α2, interacts with collagens and extracellular matrix	4313 ± 2178	4.17 × 10^–8^
CD49c	Integrin subunit α3, interacts with fibronectin, laminin, collagen, epiligrin, thrombospondin and CSPG4	12,354 ± 6447	1.45 × 10^–9^
CD49e	Integrin subunit α5, interacts with fibronectin and fibrinogen, and required for PLA2G2A	4434 ± 3870	2.76 × 10^–5^
CD54	Intercellular adhesion molecule 1, binds to integrins of type CD11a/CD18, or CD11b/CD18	16,127 ± 10,372	5.25 × 10^–8^
CD55	Decay accelerating factor, binding to C4b and C3b to prevent the formation of C4b2a and C3bBb	5019 ± 3155	3.04 × 10^–7^
CD57	β-1,3-Glucuronyltransferase 1, a key enzyme in a glucuronyl transfer reaction for the biosynthesis of the carbohydrate epitope HNK-1	2670 ± 1646	1.03 × 10^–5^
CD59	A potent inhibitor of the complement membrane attack complex, binds C8 and/or C9	25,835 ± 15,350	1.00 × 10^–8^
CD63	A member of the tetraspanin family, for the regulation of cell development, activation, growth, and motility	1497 ± 756	8.00 × 10^–4^
CD71	Transferrin receptor, for cellular iron uptake by the process of receptor-mediated endocytosis	4880 ± 4692	6.82 × 10^–5^
CD73	5’-Nucleotidase Ecto, for the conversion of extracellular nucleotides to membrane-permeable nucleosides	4737 ± 1720	4.17 × 10^–10^
CD81	A member of the tetraspanin family, for the regulation of cell development, activation, growth, and motility	7437 ± 3632	1.46 × 10^–9^
CD90	Thy-1 cell surface antigen, a member of the Ig superfamily, for cell adhesion and cell communication	6374 ± 5550	1.37 × 10^–5^
CD98	Large neutral amino acid transporter (SLC3A2 and SLC7A5), for transport of amino acids (i.e. valine, leucine, isoleucine, tryptophan, tyrosine, phenyalanine)	2743 ± 2453	1.66 × 10^–4^
CD147	Basigin, a member of the Ig superfamily, for mannose binding and is required for glucose transport	7006 ± 3790	9.66 × 10^–9^
CD151	A member of the tetraspanin family, for the regulation of cell development, activation, growth, and motility	1962 ± 1378	2.35 × 10^–4^
CD200	A member of the Ig superfamily (OX2), interacts with CD200 receptor on myeloid cells for immunosuppression	4098 ± 1970	3.49 × 10^–8^
CD201	Protein C receptor for activated protein C, a serine protease activated by and involved in the blood coagulation pathway	3674 ± 2628	5.61 × 10^–6^
β2-mic	β2 microglobulin (B2M), the light chain of MHC class 1 for the presentation of peptide antigens to the immune system	7042 ± 5835	6.15 × 10^–6^
EGFR	Epidermal growth factor receptor for EGF-mediated cell proliferation	2384 ± 757	1.26 × 10^–6^
HLA-A,B.C	Major MHC class I, the heavy chain of the class I bound to B2M for activation of CD8 T cells	8725 ± 5722	1.35 × 10^–7^
HLA-A2	A HLA serotype (HLA-A*02) within the HLA-A	9715 ± 6261	5.61 × 10^–6^
	Low-moderate level group		
CD31	Platelet and endothelial cell adhesion molecule 1 (PECAM1) for cell adhesion of leukocyte migration	1207 ± 1162	0.0131
CD45RO	An isoform of CD45, a marker of memory T cells	1326 ± 1084	0.0055
CD49a	Integrin subunit α1, a cell-surface receptor for collagen and laminin	1459 ± 1326	0.0042
CD49f	Integrin subunit α6, interacts with ECM including members of the laminin family	1140 ± 897	0.0122
CD50	Intercellular adhesion molecule 3 (ICAM3), binding to the leukocyte adhesion LFA-1 protein	1322 ± 1031	0.005
CD58	A member of the Ig superfamily, a ligand of CD2 of T cells in adhesion and activation of T cells	1748 ± 1500	0.0012
CD61	Integrin subunit β3, associated with CD51	1880 ± 2387	0.006
CD105	Endoglin (ENG), a component of the transforming growth factor β receptor complex	1354 ± 2407	0.0478
CD164	Sialomucin, for the regulation of the cell proliferation, adhesion and migration of hematopoietic progenitor cells	1565 ± 1076	0.0012
CD166	Activated leukocyte cell adhesion molecule (ALCAM), binding to CD6 of T cells in the processes of cell adhesion and migration	1307 ± 491	0.002
	Heterogenous level group		
CD43	Sialophorin, for antigen-specific activation of T cells	2242 ± 2917	0.0043
CD45	Protein tyrosine phosphatase receptor type C (PTPRC), an essential regulator of T- and B-cell antigen receptor signaling	1330 ± 1537	0.0003
CD49d	Integrin subunit α4, for cell surface adhesion and signaling	1451 ± 2365	0.0318
CD51/CD61	Integrin αv/β3, a receptor for vitronectin, cytotactin, fibronectin, fibrinogen, laminin, matrix metalloproteinase-2, etc	1016 ± 1139	0.0367
CD66b	Carcinoembryonic antigen cell adhesion molecule 8 (CEACAM8) for heterophilic cell–cell adhesion	1196 ± 1373	0.0202
CD66f	Pregnancy-specific β1-glycoprotein (PSG1), a member of the Ig superfamily	1961 ± 2092	0.0021
CD72	A signaling receptor for cell adhesion, and B-cell proliferation and differentiation	1785 ± 2576	0.0125
CD74	HLA-DR antigens-associated invariant (γ) chain, a receptor for the cytokine macrophage migration	1105 ± 1389	0.0332
CD95	Fas cell surface death receptor for regulation of apoptosis	1675 ± 2343	0.0127
CD142	Coagulation factor 3 (F3), a receptor for the coagulation factor VII for initiating the blood coagulation cascades	1582 ± 1511	0.0033
CD158b	Killer cell Ig-like receptor for the inhibitory signal on NK cell lytic activity upon interaction with HLA C	1552 ± 2922	0.0459
CD321	F11 receptor, an important regulator of tight junction assembly in epithelia, and a ligand for the integrin LFA1 in leukocyte transmigration	1386 ± 1536	0.0100
Integrin β7	Pair with CD49d to form the heterodimeric integrin receptor, for signaling from the ECM	2619 ± 4690	0.0201

*SD* standard deviation

*p value: statistical difference between the staining (MFI) with anti-target protein antibody and that with isotype control antibody

**Table 2:** The absence of some common MSC markers on the pMSCs

Protein	Function (www.genecards.org)	MFI (mean ± SD, n = 6)	*p* value*
CD106	Vascular cell adhesion molecule 1 on cytokine-activated endothelium	−72.83 ± 447.42	0.5317
CD146	Melanoma cell adhesion molecule (MCAM), play a role in cell adhesion	−109.33 ± 1232.93	0.5464
CD140b	Platelet derived growth factor receptor 1(beta) for the growth of cells of mesenchymal origin	1260.33 ± 2895.7	0.1059
CD271	Nerve growth factor receptor for differentiation and survival of specific neural cells and the response of adipose and muscle to insulin	−5.67 ± 523.04	0.6717
Disialogangli GD2	A disialoganglioside, promotes malignant phenotypes related to cell proliferation, migration, and invasion of tumor cells	683.67 ± 1054.02	0.1730
SSEA-1	Fucosyltransferase 4, transfers fucose to N-acetyllactosamine polysaccharides to generate fucosylated carbohydrate structure	1361.33 ± 3635.57	0.1403
SSEA-3	Stage-specific embryonic antigen 3, expressed along with SSEA04, TRA-1-60, and TRA-1-8	−62 ± 632.65	0.5662
SSEA-4	A glycosphingolipid expressing along with SSEA-3, TRA-1-60 and TRA-1-81 in embryonic stem cells, embryonal carcinoma cells and induced pluripotent stem cells	94.5 ± 586.3	0.8952

*SSEA* Stage-specific embryonic antigen, *SD* standard deviation

*p value: statistical difference between the staining (MFI) with anti-target protein antibody and that with isotype control antibody

The high level group consisted of 28 cell surface proteins [CD9, CD26, CD29, CD42a, CD44, CD46, CD47, CD49b, CD49c, CD49e, CD54, CD55, CD57, CD59, CD63, CD71, CD73, CD81, CD90, CD98, CD147, CD151, CD200, CD201, β2-microglobin, epithelial growth factor (EGF) receptor, and human leukocyte antigen (HLA) class I (HLA-A,B,C) including HLA-A2 in 5 of 6 patients] (Table 1). Among these strongly positive-stained cell surface proteins, the expression of CD44, CD46, CD49c, CD59, CD73, CD81 and CD147 was found at the highest levels, and they could consider as the common markers for this MSCs population, which was indicated by the extremely low *p* values (≤ 1.0 × 10^–8^) as compared with isotype antibody controls (Table 1).

In the low to moderate group, there were 10 cell surface proteins (CD31, CD45RO, CD49a, CD49f, CD50, CD58, CD61, CD105, CD164, and CD166) (Table 1). In this group, the staining intensity of target protein expression was positive but was at a low level in some samples as compared with the isotype controls (*p* < 0.05, Table 1), suggesting that these proteins might be considered as weak markers for this population of MSCs.

In the heterogeneous group, the MFI of CD43, CD45, CD49d, CD51/CD61, CD66b, CD66f, CD72, CD74, CD95, CD142, CD158b, CD321 and integrin β7 was statistically higher than those of the isotype controls (*p* < 0.05), but their expression was heterogenous, in which the negative expression was found in one to three out of six samples (Table 1). These data may imply that the expression of these proteins on pMSCs was donor-dependent, and they probably are not specific for this population of MSCs when the sample size is larger than 6 patients.

In addition to CD73, CD90 and CD105, other cell surface proteins have been identified as MSCs markers for MSCs from different sources such as STRO-1, CD271, SSEA-4, CD146, GD2, CD106 and CD140b [21–23], which however were found negative in pMSCs (Table 2). Furthermore, in general, MSCs can be defined as mesenchymal, so that they are negative in the expression of the specific hematopoietic cell surface markers. The data from the present study showed that except heterogeneous expression of CD45, the expression of all of other specific hematopoietic cell or MSC negative markers (CD11b, CD14, CD19, CD34, CD40, CD79b, CD80, CD86) was not found on pMSCs as well (Table 3).

**Table 3:** The lack of the expression of some typical hematopoietic markers

Protein	Function (www.genecards.org)	MFI (mean ± SD, n = 6)	*p* value*
CD11b	Integrin subunit αM, a component of leukocyte-specific integrin (macrophage receptor 1, Mac-1)	45.27 ± 818.83	0.7955
CD14	A myeloid cell-specific leucine-rich glycoprotein, preferentially expressed on monocytes/macrophages	67.0 ± 363.8	0.8269
CD19	A member of the Ig gene superfamily, restricted to B cells	717.0 ± 580.47	0.1063
CD34	A hematopoietic progenitor cell antigen, for early hematopoiesis	577.67 ± 1087.88	0.2743
CD40	A receptor on antigen-presenting cells for stimulation of T cells	356.17 ± 599.61	0.5383
CD79b	A B cell antigen receptor, in cooperation with CD79a for signaling of B-cell receptor	562.33 ± 455.96	0.2215
CD80	A receptor on antigen-presenting cells for CD28 or CTLA-4 for T cell activation and cytokine production	463.83 ± 628.08	0.3612
CD86	A receptor on antigen-presenting cells for CD28 or CTLA-4 for T cell activation and cytokine production	-407.33 ± 659.40	0.1300
HLA-DR	MHC class II cell surface receptor, a ligand for the TCR	506.83 ± 1618.85	0.4333

*SD* standard deviation

*p value: statistical difference between the staining (MFI) with anti-target protein antibody and that with isotype control antibody

### Comparison of cell surface protein phenotype of pMSCs with other types of MSCs

To further characterize the cell surface protein phenotype of pMSCs, the expression profiling of cell surface proteins of pMSCs was compared with those of well-characterized BM-MSCs and AT-MSCs. The data of the comprehensive expression of cell surface proteins on either BM-MSCs (83 proteins) or AT-MSCs (112 proteins) were obtained from published literature [14, 21, 24, 25], which were used as references to compare the cell surface protein profiling (112 proteins) of pMSCs that was determined in both our previous studies [17–20] and the present study (S2 Table). Table 4 showed that the MSCs from all of these three different sources shared the positive expression of 28 cell surface proteins (CD9, CD29, CD44, CD46, CD47, CD49a-e, CD51, CD54, CD58, CD59, CD61, CD63, CD71, CD73, CD81, CD90, CD95, CD98, CD105, CD147, CD151, CD166, CD200, and HLA class I), and the negative expression of 19 proteins (CD1a, CD3, CD4, CD5, CD11a, CD11b, CD14, CD15, CD18, CD19, CD25, CD38, CD56, CD62E, CD62P, CD123, CD144, CD178, and HLA-DR), the typical hematopoietic cell markers.

**Table 4:** The difference of cells surface protein profiling between MSCs from BM, AT, and peritoneal dialysis effluent

Cell surface protein (synonym)	BM-MSCs*	AT-MSCs**	pMSCs
CD1a (HTA-1)	–	–	–
CD3 (T cell co-receptor)	–	–	–
CD4 (T4)	–	–	–
CD5 (Ly-1)	–	–	–
CD11a (Integrin αL, ITGAL)	–	–	–
CD11b (Integrin αM, ITGAM)	–	–	–
CD14	–	–	–
CD15 (LewisX)	–	–	–
CD18 (Integrin β2, ITGB2)	–	–	–
CD19	–	–	–
CD25 (IL-2R)	–	–	–
CD38 (ADP-RC)	–	–	–
CD56 (NCAM)	–	–	–
CD62E (E-selectin)	–	–	–
CD62P (P-selectin)	–	–	–
CD123 (IL-3R-α)	–	–	–
CD144 (Cadherin-5)	–	–	–
CD178 (FasL)	–	–	–
HLA-DR	–	–	–
CD9 (Tetraspanin-29)	+	+	+
CD29 (Integrin β1, ITGB1)	+	+	+
CD44 (H-CAM)	+	+	+
CD46	+	+	+
CD47	+	+	+
CD49a (Integrin α1)	+	+	+
CD49b (integrin α2)	+	+	+
CD49e (integrin α5)	+	+	+
CD58 (LFA-3)	+	+	+
CD59 (MIRL)	+	+	+
CD61 ((integrin β3)	+	+	+
CD63 (LIMP)	+	+	+
CD73 (ecto-5’-nuclotidase)	+	+	+
CD81	+	+	+
CD90 (Thy-1)	+	+	+
CD98	+	+	+
CD147	+	+	+
CD151	+	+	+
CD200	+	+	+
HLA-A,B,C (HLA class I)	+	+	+
CD49c (integrin α3)	+	+ /Varies	+
CD51 ((integrin αv)/61	+ (CD51 only)	+	+ /Varies
CD95 (Fas)	+	+	+ /Varies
CD105 (endoglin)	+	+	+ /Varies
CD166 (ALCAM)	+ /varies	+	+
CD49d (integrin α4)	±	+	+ /Varies
CD54 (ICAM-1)	±	+	+
CD71 (transferrin receptor)	±	+	+
CD99	+	+	–
CD140a (PDGFRα)	+	+	–
CD140b (PDGFRβ)	+	+	–
CD340 (HER-2/erb-2)	+	+	–
CD10 (CALLA)	±	+	–
CD13 (APN)	±	+	–
CD34	±	+ /Varies	–
CD36	±	+ /Varies	–
CD146 (MCAM)	+	–/ +	–
CD271 (NGFR)	+	–/ +	–
GD2	+ ^#^	–/ + ^#^	–
CD50 (ICAM-3)	–	–	+
CD62L (L-selectin)	+	–	–
CD104 (integrinβ4)	+	–	–
CD109	+	–	–
CD119 (IFNγR)	+	–	–
CD120a (TNFIR)	+	–	–
CD120b (TNFIIR)	+	–	–
CD121a (IL-1R)	+	–	–
CD124 (IL-4R)	+	–	–
CD126 (IL-6R)	+	–	–
CD127 (IL-7R)	+	–	–
CD172	+ (CD172a)	–(CD172b)	–(CD172b)
CD221	+	–	–
SSEA-3	+	–	–
STRO-1	+	–	–^##^
CD112	+	–/Varies	–
CD106 (VCAM1)	+ /Varies	–	–
CD33 (gp67)	±	–	–
CD117 (c-kit)	±	–	–
SSEA-4	±	–	–
CD102 (ICAM-2)	±	–/Varies	–
CD49f (integrin α6)	+	–/Varies	+
CD97	+	–/Varies	+ /Varies
CD31	±	–	+
CD45 (LCA)	±	–	±
CD26 (ADA-BP)	NR	+	+
CD55	NR	+	+
CD201	NR	+	+
β2-mic	NR	+	+
EGFR	NR	+	+
CD142	NR	+	+ /Varies
CD164	NR	+	+ /Varies
SSEA-1	NR	–	–
CD91	NR	+	–
CD99R	NR	+	–
CD165	NR	+	–
CD183	NR	+	–
CD209	NR	+	–
CD227	NR	+	–
CD273	NR	+	–
CD39	NR	+ /Varies	–
CD130	NR	+ /Varies	–
CD141	NR	+ /Varies	–
CD42a	NR	–	+
CD45RO	NR	–	+
CD57	NR	–	+
CD43	NR	–	+ /Varies
CD66b	NR	–	+ /Varies
CD66f	NR	–	+ /Varies
CD72	NR	–	+ /Varies
CD74	NR	–	+ /Varies
CD158b	NR	–	+ /Varies
CD321	NR	–	+ /Varies
Integrinβ7	NR	–	+ /Varies

^*^Data of BM-MSCs are derived from literature [14, 21–24]

**Data of AT-MSCs from previous publications [21–23, 25]

^#^Positive expression of GD2 on both BM-MSCs and AT-MSCs [26]

^##^Negative expression of STRO-1 on pMSCs [17–20]. + and − : the presence or absence of a given protein expression, respectively, ± : both positive and negative reported within this MSC population in different studies, “varies”: various levels from low or negative to high among the samples tested, “*NR*” not reported. *BM*: bone marrow; *AT* adipose tissue

In comparison with the expression of remaining proteins (36 out 83 proteins) in BM-MSCs, except positive expression of CD49f, CD97, CD31, and CD45 in both, the expression of 32 proteins (38.55%) was different between BM-MSCs and pMSCs. The positive expression of 31 proteins [CD99, CD140a-b, CD340, CD146, CD271, GD2, CD62L, CD104, CD109, CD119, CD120a-b, CD121a, CD124, CD126, CD127, CD172, CD221, SSEA-3/4, STRO-1, CD112, CD106, CD33, CD117, CD102, CD10, CD13, CD34, and CD36] was found on the BM-MSCs but not on the pMSCs, and CD50 was negative on the BM-MSCs but positive on the pMSCs (Table 3).

The cell surface phenotype of AT-MSCs [25] were directly compared with that of pMSCs from the present study (S2 Table) to reveal any differences between these two MSC populations using the same panel of antibodies. AT-MSCs were positive in the expression of 18 markers [CD10, CD13, CD34, CD36, CD39, CD91, CD99, CD99R, CD130 (IL-6Rβ), CD140a (PDGFRα), CD140b (PDGFRβ), CD141 (thrombomodulin), CD165, CD183 (CXCR3), CD209, CD227, CD273 (PD-L2), and CD340 (HER2)], which were negative on the pMSCs (Table 4). On the other hand, the positive expression of 7 markers [CD31 (PCAM1), CD42a, CD45RO, CD49f, CD50 (ICAM3), CD57, and Integrin β7) and probably positivity of additional 9 markers [CD43, CD45, CD66b, CD66f, CD72, CD74, CD97, CD158b (KIR2DL3), and CD321] were found on the pMSCs, which were not seen on AT-MSCs (Table 4). Hence, the difference between AT-MSCs and pMSCs was found in the expression of 34 out of 242 (approximately 14.1%) of cell surface proteins.

## Discussion

PD is an effective kidney replacement therapy for fluid management of patients with end-stage kidney disease; however, the side effect of bioincompatible PD solutions causes peritoneal membrane injury and inflammation, which eventually result in ultrafiltration failure [27, 28]. Numerous studies have documented that MSCs have a wide spectrum of anti-inflammatory and immunomodulatory activities and have been tested in the treatment of various tissue or organ injuries, immunologic diseases and aging frailty [29, 30]. We for the first time isolated MSCs from otherwise discarded PD effluent [17], and administration of these PD effluent-derived MSCs (pMSCs) prevents the bioincompatible PD solution-caused PM injury in a rat model of PD [19, 20], suggesting the potential of using autologous MSCs from PD effluent for the preservation of peritoneal membrane structure and function in PD patients. However, proteins on the cell surface have different distinct functions for a given cell and are often used as lineage-specific markers. To further characterize pMSCs, the present study is designed to verify the cell surface protein phenotype of pMSCs using flow cytometric analysis with a panel of 242 antibodies to cell surface proteins (S2 Table), the same panel of the antibodies has been used to identify the phenotype of AT-MSCs [25]. As compared with the cell surface phenotypes of BM-MSCs and AT-MSCs in the literature [14, 21, 22, 24, 25] (Table 4), MSC originated from these three different locations share the positive expression of a panel of 28 cell surface proteins, and 18 of them are expressed at a high level (the high level group) on pMSCs. Functional enrichments analysis using STRING database (https://string-db.org) shows that the tissue expression of these 28 proteins with strength of over 2.0 at the top of the list are as follows: “Mesenchymal stromal cell” (Strength: 2.88, and false discovery rate: 7.88 × 10^–6^), “Chondroblast” (Strength: 2.88, and false discovery rate: 7.88 × 10^–6^), “adipose-derived stem cell” (Strength: 2.88, and false discovery rate: 0.00074), and “Mesenchymal stem cell” (Strength: 2.35, and false discovery rate: 7.47 × 10^–5^), suggesting that these proteins as a group may be represented as specific markers for MSCs. The functions of these proteins (www.genecards.org) can be found as: (1) Members of the tetraspanin family (CD9, CD63, CD81, and CD151) for cell differentiation, activation, growth and motility or adhesion, (2) members of the integrin family (CD29, CD49, and CD51/61) for cell adhesion, (3) partners of the tetraspanin and/or integrin members (CD44, CD47, CD58, CD90 and CD147) for cell–cell interaction and cell adhesion, and (4) regulatory factors for inactivation of complements (CD46, CD59), natural killer (NK) cell-mediated cytotoxicity (CD161) and pro-inflammatory immune response (CD200), which together may reflect their multipotency of differentiation and immune modulation. In addition, the nonimmunogenic properties of these MSCs including pMSCs are indicated by the lack of MHC class II antigens (HLA-DR) and a panel of hematopoietic cell marker CD1a, CD3, CD4, CD5, CD11a, CD11b, CD14, CD15, CD18, CD19, CD25, CD38, CD56, CD62P, CD144, and CD178, and the costimulatory molecules CD40, CD80, and CD86. Thus, like BM-MSCs and AT-MSCs, these characteristics make pMSCs promising candidates for new therapeutic strategies in transplantation and regenerative medicine.

According to Dominic et al. [1], expression of CD105 is one of essential markers to confirm the phenotype of MSCs. Our previous examination of CD105 (Endoglin, a component of the TGF-β receptor) has showed the negative expression of this protein on pMSCs [17–20], but the present study shows that the expression of CD105 is positive in four out of six donor samples (S1 Table), and the MFI of CD105 staining is statistically significant higher than that of isotype controls in this group of donors (Table 1). These data indicate that the discrepancy in the expression of CD105 on pMSCs between our studies may be due to different donors or different antibodies used between these examinations.

Furthermore, although the data for a comparison of all of the cell surface proteins between BM-MSCs, AT-MSCs and pMSCs are not available, at least there are 16 cell surface proteins expressing on BM-MSCs but not on either AT-MSCs or pMSCs (Table 4). These cell surface proteins noticeably include a group of receptors of leukocyte-producing cytokines (IFNγR1, TNFRSF1, IL-1R1, IL-4R, IL-6Rα, and IL-7R), implying that as compared with BM-MSCs, both AT-MSCs and pMSCs that are deficient in these receptors may become less affected by activated leukocytes, which remains further investigation.

Using the same panel of antibodies (BD Lyoplate™, BD Biosciences) to compare the cell surface phenotype of AT-MSCs [25] and the pMSCs presented by the present study (Table S2), these two MSC populations are different in the positive expression of 34 out of 242 cell surface proteins (14.1%), 18 including PDGFR (CD140) positive on AT-MSCs and 16 positive on pMSCs (Table 4), suggesting that AT-MSCs and pMSCs may not be the same lineage, or the pMSCs may be not originated from the adipose tissues of peritoneal cavity. The functional enrichments analysis using the STRING database reveals that the network of 18 cell surface proteins on AT-MSCs is mainly involved in “Platelet-derived growth factor-activated receptor activity” (Strength: 2.91, false discovery rate: 0.0040) and “Vascular endothelial growth factor binding” (strength: 2.54, false discovery rate: 0.0092), whereas the 16 proteins on pMSCs are mainly associated with “Other semaphorin interaction” (strength: 2.17, false discovery rate: 0.0430) and “Cell surface interactions at the vascular wall” (strength: 1.85, false discovery rate: 10 × 10^–9^), which may reflect a difference of the microenvironment where they survive and proliferate. However, it is still possible that 14.1% of differences between pMSCs and AT-MSCs in Table 4 may be due to the demographic disparities of samples and/or experimental errors between laboratories, so that the difference in cell surface markers between these two types of MSCs needs to be confirmed from the same donors by the same group. Other studies for verifying the pMSC lineage or stem cell niche may include the investigation of epigenetic memory of pMSCs as compared with AT-MSCs in the differentiation to adipocytes or others as described previously [16].

The obstacles to clinical use of autologous pMSCs for prevention of PM injury in PD patients will be the same as discussed recently [6]. To further develop an effective pMSCs-based therapy for PD patients, the following steps are needed as follows: (1) to identify the functional marker(s) of pMSCs that will be used as standard criteria for quality control in pMSCs preparation, (2) to optimize the culture conditions for large-scale expansion of pMSCs, and (3) to investigate the mechanisms underlying the therapeutic activities of pMSCs specifically for peritoneal cavity and any in vitro expansion-derived potential risks such as tumorigenesis of pMSCs.

One has to acknowledge the following limitations of this study: (1) the sample size might be too small (*n* = 6), which could reduce the power of the conclusion or increase the margin of error. For example, the statistical significance of some proteins in the heterogeneous group (Table 1) probably become not significant when more samples are included, (2) the purity of cultured MSCs from each patient after 4–7 passages was not checked, which may affect the MFI value in flow cytometric analysis, and (3) the specificity of FACS analysis of target proteins were not verified or confirmed by alternative methods such as Western blotting analysis using different antibodies.

## Conclusions

Data from the present study reveal a comprehensive cell surface protein profiling of pMSCs that were isolated from otherwise discarded PD effluent. Specifically, progress has been made in the comparison of the cell surface proteins between BM-MSCs, AT-MSCs and pMSCs, which indicates that pMSCs share MSCs-specific markers with BM-MSCs or AT-MSCs, but the difference of cell surface proteins among them may imply that the biological characteristics of pMSCs including their lineage and functions are different from those of AT-MSCs and BM-MSCs. The knowledge from the present study may be required for further development of pMSCs-based therapy in the treatment of PD-induced PM injury in patients who receive maintenance PD.

### Supplementary Information

Below is the link to the electronic supplementary material.

Supplementary file1 (DOCX 13 KB)

Supplementary file2 (DOCX 44 KB)

## Data Availability

All data generated or analysed during this study are included in this published article and its supplementary information files. Materials are available upon request.

## Abbreviations

AT: Adipose tissue
BM: Bone marrow
CD: Cluster of differentiation
DMEM: Dulbecco’s modified Eagle’s medium
EDTA: Ethylenediaminetetraacetic acid
EGF: Epithelial growth factor
EPS: EDTA-containing BD pharmingen stain buffer
FBS: Fetal bovine serum
HLA: Human leukocyte antigen
IFN: Interferon
IL: Interleukin
MFI: Mean fluorescence intensity
MSCs: Mesenchymal stromal cells
PBS: Phosphate buffered saline
PD: Peritoneal dialysis
PM: Peritoneal membrane
pMSCs: Peritoneal mesenchymal stromal cells
SSEA: Stage-specific embryonic antigen
TNF: Tumor necrosis factor
UC: Umbilical cord
