# Supplementary material for: Comprehensive cell surface protein profiling of human mesenchymal stromal cells from peritoneal dialysis effluent and comparison with those from human bone marrow and adipose tissue
Source: Hum Cell. 2023 Aug 21;36(6):2259–69. doi: 10.1007/s13577-023-00971-x (PMC10587256; doi:10.1007/s13577-023-00971-x)
Supplement: Supplementary file 1 — Supplementary file1 (DOCX 13 KB) [file 13577_2023_971_MOESM1_ESM.docx]

S1 Table. The demographic Information of donors

| Donor number | Age (yr) | Gender (F/M) | NIH ethnicity categories | Time on PD (wk) | PD solution |
| --- | --- | --- | --- | --- | --- |
| 1 | 35 | F | White | 1 | Dianeal |
| 2 | 59 | M | White | 1 | Dianeal |
| 3 | 61 | M | Asian | The first PD | Dianeal |
| 4 | 81 | M | Asian | The first PD | Physioneal |
| 5 | 35 | F | Asian | 3 | Physioneal |
| 6 | 54 | M | Chinese | 1 | Dianeal |

The PD effluents were collected under the protocol H15-02466 approved by the Clinical Research Ethics Board at the University of British Columbia and Fraser Health Authority (BC, Canada).
