# Supplementary material for: Comprehensive cell surface protein profiling of human mesenchymal stromal cells from peritoneal dialysis effluent and comparison with those from human bone marrow and adipose tissue
Source: Hum Cell. 2023 Aug 21;36(6):2259–69. doi: 10.1007/s13577-023-00971-x (PMC10587256; doi:10.1007/s13577-023-00971-x)
Supplement: Supplementary file 2 — Supplementary file2 (DOCX 44 KB) [file 13577_2023_971_MOESM2_ESM.docx]

S2 Table. The summary of mean fluorescent intensity (MFI) of 242 cell surface proteins on pMSCs from 6 donors

| Protein | #1 | #2 | #3 | #4 | #5 | #6 | Mean | SD | *p* value |
| --- | --- | --- | --- | --- | --- | --- | --- | --- | --- |
| CD1a | 111.25 | -104.75 | -108.5 | 516.25 | 201 | 1193.75 | 301.5 | 494.3 | 0.6387 |
| CD1b | 124.25 | 238.25 | -288.5 | 171.25 | -144 | 1240.75 | 223.67 | 537.55 | 0.8094 |
| CD1c | -168.75 | 23.25 | -196.5 | -510.75 | -323 | -492.25 | -278 | 205.64 | 0.2116 |
| CD2 | 348.25 | -344.75 | -108.5 | 101.25 | -62 | -506.25 | -95.33 | 305.86 | 0.4812 |
| CD3 | 620.25 | 950.25 | -286.5 | -124.75 | 15 | -397.25 | 129.5 | 537.17 | 0.9748 |
| CD4 | -6.75 | 1984.25 | -20.5 | -209.75 | -44 | -142.25 | 260.17 | 848.26 | 0.7465 |
| CD4v4 | 752.25 | 422.25 | -186.5 | 257.25 | -243 | -337.25 | 110.83 | 434.56 | 0.9305 |
| CD5 | 1274.25 | 674.25 | -128.5 | -643.75 | -172 | -337.25 | 111.17 | 718.11 | 0.9350 |
| CD6 | 634.25 | 24.25 | -290.5 | 90.25 | -322 | -455.25 | -53.17 | 397.33 | 0.5670 |
| CD7 | -64.75 | 26.25 | -182.5 | -762.75 | -317 | -296.25 | -266.17 | 276.83 | 0.2273 |
| CD8a | 1208.25 | 1449.25 | -34.5 | 652.25 | -93 | -509.25 | 445.5 | 782.81 | 0.4052 |
| CD8b | 781.25 | 1070.25 | -193.5 | 325.25 | 314 | 930.75 | 538 | 474.82 | 0.2500 |
| CD9 | 3004.25 | 1412.25 | 6125.5 | 3622.25 | 1918 | 1047.75 | 2855 | 1872.58 | 1.03E-05 |
| CD10 | 295.25 | 1337.25 | -92.5 | 74.25 | -277 | -403.25 | 155.67 | 629.83 | 0.9654 |
| CD11a | 425.25 | 415.25 | 150.5 | -251.75 | -129 | -154.25 | 76 | 298.35 | 0.8466 |
| CD11b | 1049.25 | 948.25 | 15.5 | -1041.75 | -220 | -480.25 | 45.17 | 818.83 | 0.7955 |
| CD11c | 370.25 | 151.25 | -6.5 | 648.25 | -234 | -296.25 | 105.5 | 362.21 | 0.9171 |
| CD13 | 1341.25 | 6311.25 | 302.5 | -294.75 | -298 | -443.25 | 1153.17 | 2612.12 | 0.1139 |
| CD14 | 752.25 | -172.75 | -176.5 | 178.25 | -6 | -173.25 | 67 | 363.80 | 0.8269 |
| CD15 | 232.25 | 736.25 | 84.5 | 96.25 | -464 | -421.25 | 44 | 445.77 | 0.7766 |
| CD15s | 1244.25 | -237.75 | 28.5 | 71.25 | -502 | -584.25 | 3.33 | 663.91 | 0.6991 |
| CD16 | 987.25 | 552.25 | -97.5 | -234.75 | -295 | -424.25 | 81.33 | 560.64 | 0.8647 |
| CD18 | 1909.25 | -71.75 | -184.5 | 231.25 | -363 | -315.25 | 201 | 863.33 | 0.8705 |
| CD19 | 318.25 | 801.25 | -11.5 | 1649.25 | 535 | 1009.75 | 717 | 580.47 | 0.1063 |
| CD20 | 174.25 | 927.25 | -16.5 | -197.75 | -33 | N/A | 170.85 | 442.91 | 0.9344 |
| CD21 | 289.25 | 1195.25 | -176.5 | -477.75 | -272 | -62.25 | 82.67 | 601.42 | 0.8688 |
| CD22 | 431.25 | 1095.25 | -101.5 | 152.25 | -64 | -344.25 | 194.83 | 512.62 | 0.8741 |
| CD23 | 12.25 | 21.25 | 245.5 | -427.75 | -315 | -100.25 | -94 | 244.99 | 0.4809 |
| CD24 | -12.75 | 874.25 | -158.5 | -174.75 | 43 | -174.25 | 66.17 | 406.47 | 0.8260 |
| CD25 | 609.25 | 852.25 | 5.5 | 108.25 | -400 | -418.25 | 126.17 | 519.36 | 0.9670 |
| CD26 | 4270.25 | 3798.25 | 5116.5 | 4923.25 | 1705 | 1565.75 | 3563.17 | 1565.91 | 5.04E-08 |
| CD27 | 91.25 | 948.25 | 317.5 | 819.25 | -275 | -298.25 | 267.17 | 532.1 | 0.7134 |
| CD28 | 696.25 | 940.25 | -182.5 | -574.75 | -236 | -54.25 | 98.17 | 588.78 | 0.9035 |
| CD29 | 3874.25 | 4387.25 | 751.5 | 3648.25 | 476 | 985.75 | 2353.83 | 1793.73 | 0.0001 |
| CD30 | 458.25 | 462.25 | -146.5 | 862.25 | 38 | -429.25 | 207.5 | 472.43 | 0.8441 |
| CD31 | 884.25 | 285.25 | 158.5 | 3255.25 | 839 | 1816.75 | 1206.5 | 1162.38 | 0.0131 |
| CD32 | 1142.25 | 554.25 | 345.5 | 1492.25 | -67 | -136.25 | 555.17 | 652.98 | 0.2465 |
| CD33 | 476.25 | 1182.25 | 171.5 | 2223.25 | -9 | -352.25 | 615.33 | 943.31 | 0.2176 |
| CD34 | 574.25 | 415.25 | 175.5 | 2690.25 | -3 | -386.25 | 577.67 | 1087.88 | 0.2743 |
| CD35 | 311.25 | 805.25 | 94.5 | -332.75 | -80 | -467.25 | 55.17 | 462.91 | 0.8025 |
| CD36 | 581.25 | 1602.25 | 139.5 | -888.75 | -214 | -225.25 | 165.83 | 853.92 | 0.9453 |
| CD37 | 431.25 | 935.25 | -225.5 | 571.25 | -203 | 753.75 | 377.17 | 488.60 | 0.4908 |
| CD38 | 2442.25 | 424.25 | 684.5 | 580.25 | -40 | -93.25 | 666.33 | 926.94 | 0.1718 |
| CD39 | 1398.25 | 72.25 | -74.5 | 209.25 | 23 | 1137.75 | 461 | 637.11 | 0.3663 |
| CD40 | 1355.25 | 669.25 | -36.5 | 434.25 | 5 | -290.25 | 356.17 | 599.61 | 0.5383 |
| CD41a | 941.25 | 796.25 | -100.5 | 317.25 | -103 | -399.25 | 242 | 538.39 | 0.7687 |
| CD41b | 632.25 | 721.25 | -100.5 | 760.25 | 213 | -260.25 | 327.67 | 442.03 | 0.5823 |
| CD42a | 1731.25 | 350.25 | 353.5 | 5553.25 | 1638 | 2606.75 | 2038.83 | 1929.80 | 0.0009 |
| CD42b | 851.25 | 818.25 | -84.5 | 3618.25 | -116 | 153.75 | 873.5 | 1410.82 | 0.1013 |
| CD43 | 730.25 | 1118.25 | 84.5 | 6311.25 | 5561 | -356.25 | 2241.5 | 2916.45 | 0.0043 |
| CD44 | 51022.25 | 18419.25 | 22586.5 | 38586.25 | 31226 | 17187.75 | 29838 | 13192.66 | 2.48E-11 |
| CD45 | 1050.25 | 469.25 | 255.5 | 3725.25 | 2668 | -191.25 | 1329.5 | 1537.23 | 0.0003 |
| CD45RA | 430.25 | 1603.25 | 15.5 | 984.25 | -92 | -162.25 | 463.17 | 703.16 | 0.3700 |
| CD45RB | 914.25 | 201.25 | 98.5 | 283.25 | -307 | -69.25 | 186.83 | 413.74 | 0.8907 |
| CD45RO | 2385.25 | 1278.25 | 177.5 | 2757.25 | 159 | 1195.75 | 1325.5 | 1083.58 | 0.0055 |
| CD46 | 13014.25 | 4195.25 | 11501.5 | 12613.25 | 8199 | 9982.75 | 9917.67 | 3318.66 | 5.53E-13 |
| CD47 | 3233.25 | 2038.25 | 2080.5 | 3168.25 | 842 | 2418.75 | 2296.83 | 881.06 | 3.79E-06 |
| CD48 | 1094.25 | 104.25 | -146.5 | 808.25 | 43 | -233.25 | 278.33 | 543.03 | 0.6901 |
| CD49a | 1352.25 | 1044.25 | 1259.5 | 4063.25 | 596 | 437.75 | 1458.83 | 1326.13 | 0.0042 |
| CD49b | 2428.25 | 2602.25 | 3628.5 | 8292.25 | 5127 | 3797.75 | 4312.67 | 2177.61 | 4.17E-08 |
| CD49c | 16695.25 | 1358.25 | 16663.5 | 14919.25 | 16874 | 7611.75 | 12353.67 | 6447.09 | 1.45E-09 |
| CD49d | 1351.25 | 1436.25 | 39.5 | 6056.25 | -53 | -127.25 | 1450.5 | 2364.90 | 0.0318 |
| CD49e | 3737.25 | 3500.25 | 3722.5 | 12076.25 | 2325 | 1244.75 | 4434.33 | 3870.06 | 2.76E-05 |
| CD49f | 928.25 | 1034.25 | 343.5 | 2366.25 | 2040 | 128.75 | 1140.17 | 897.42 | 0.0122 |
| CD50 | 1802.25 | 1282.25 | 130.5 | 2070.25 | 78 | 2570.75 | 1322.33 | 1031.38 | 0.0050 |
| CD51/61 | 2650.25 | -273.75 | 1066.5 | 2051.25 | 474 | 127.75 | 1016 | 1139.41 | 0.0367 |
| CD53 | 1727.25 | 1658.25 | -167.5 | 1083.25 | -125 | -120.25 | 676 | 919.01 | 0.1634 |
| CD54 | 21426.25 | 2322.25 | 32636.5 | 17448.25 | 10611 | 12319.75 | 16127.33 | 10371.92 | 5.25E-08 |
| CD55 | 5116.25 | 2694.25 | 4074.5 | 11246.25 | 3574 | 3409.75 | 5019.17 | 3154.75 | 3.04E-07 |
| CD56 | 1278.25 | 972.25 | 168.5 | 1231.25 | -61 | -76.25 | 585.5 | 644.40 | 0.2137 |
| CD57 | 3956.25 | 347.25 | 2229.5 | 2021.25 | 2388 | 5078.75 | 2670.17 | 1645.97 | 1.03E-05 |
| CD58 | 3670.25 | 261.25 | 2567.5 | 2965.25 | 245 | 778.75 | 1748 | 1500.48 | 0.0012 |
| CD59 | 37887.25 | 20658.25 | 49681.5 | 24494.25 | 12559 | 9731.75 | 25835.33 | 15349.76 | 1.00E-08 |
| CD61 | 1823.25 | 1252.25 | 479.5 | 6623.25 | 772 | 328.75 | 1879.83 | 2387.10 | 0.0060 |
| CD62E | 1415.25 | 1576.25 | 232.5 | 871.25 | -385 | -109.25 | 600.17 | 812.35 | 0.2168 |
| CD62L | 713.25 | 1057.25 | -29.5 | 4037.25 | 6 | -98.25 | 947.67 | 1584.17 | 0.0880 |
| CD62P | -99.75 | 1062.25 | -25.5 | 691.25 | -302 | 3033.75 | 726.67 | 1244.46 | 0.1647 |
| CD63 | 1784.25 | 2789.25 | 841.5 | 1615.25 | 738 | 1210.75 | 1496.5 | 755.53 | 0.0008 |
| CD64 | 1319.25 | 735.25 | 119.5 | 676.25 | -283 | 117.75 | 447.5 | 573.59 | 0.3804 |
| CD66 (a,c,d,e) | 1918.25 | 151.25 | 713.5 | 2586.25 | -160 | -139.25 | 845 | 1154.23 | 0.0893 |
| CD66b | 1389.25 | 1114.25 | 58.5 | 3629.25 | -275 | 1256.75 | 1195.5 | 1373.04 | 0.0202 |
| CD66f | 1228.25 | 1567.25 | 301.5 | 3956.25 | -301 | 5015.75 | 1961.33 | 2091.65 | 0.0021 |
| CD69 | 1027.25 | 1834.25 | 205.5 | 205.25 | -18 | -335.25 | 486.5 | 799.59 | 0.3479 |
| CD70 | 1576.25 | 520.25 | -198.5 | 803.25 | 544 | 3.75 | 541.5 | 628.72 | 0.2596 |
| CD71 | 2389.25 | 426.25 | 2105.5 | 13584.25 | 5561 | 5211.75 | 4879.67 | 4692.02 | 6.82E-05 |
| CD72 | 1994.25 | 460.25 | -119.5 | 6685.25 | -145 | 1835.75 | 1785.17 | 2575.49 | 0.0125 |
| CD73 | 4633.25 | 4662.25 | 4953.5 | 7645.25 | 2283 | 4246.75 | 4737.33 | 1719.51 | 4.17E-10 |
| CD74 | 2545.25 | 1901.25 | -43.5 | 2608.25 | -231 | -152.25 | 1104.67 | 1389.44 | 0.0332 |
| CD75 | 464.25 | 953.25 | 431.5 | 658.25 | -35 | 325.75 | 466.33 | 330.53 | 0.3336 |
| CD77 | 674.25 | 1131.25 | -5.5 | 296.25 | -436 | 3461.75 | 853.67 | 1387.33 | 0.1079 |
| CD79b | 634.25 | 798.25 | 485.5 | 1165.25 | 507 | -216.25 | 562.33 | 455.96 | 0.2215 |
| CD80 | 1448.25 | -189.75 | 226.5 | 1017.25 | 187 | 93.75 | 463.83 | 628.08 | 0.3612 |
| CD81 | 11385.25 | 6669.25 | 10789.5 | 9247.25 | 2797 | 3730.75 | 7436.5 | 3632.09 | 1.46E-09 |
| CD83 | 687.25 | 399.25 | 151.5 | 1235.25 | -193 | 19.75 | 383.33 | 517.01 | 0.4819 |
| CD85 | 1089.25 | 429.25 | 163.5 | 233.25 | -157 | 141.75 | 316.67 | 423.07 | 0.6034 |
| CD86 | 495.25 | -1487.75 | -82.5 | -681.75 | -304 | -383.25 | -407.33 | 659.40 | 0.1300 |
| CD87 | 355.25 | -543.75 | -257.5 | -1016.75 | -418 | -555.25 | -406 | 450.77 | 0.1166 |
| CD88 | 260.25 | -211.75 | -175.5 | -917.75 | -267 | -332.25 | -274 | 378.64 | 0.2243 |
| CD89 | 517.25 | -898.75 | -260.5 | 99.25 | -360 | -401.25 | -217.33 | 481.71 | 0.3001 |
| CD90 | 13248.25 | 2180.25 | 5274.5 | 13468.25 | 1994 | 2077.75 | 6373.83 | 5550.18 | 1.37E-05 |
| CD91 | 758.25 | 1137.25 | -20.5 | -177.75 | -191 | -280.25 | 204.33 | 594.11 | 0.8547 |
| CDw93 | 540.25 | 2244.25 | -144.5 | -88.75 | -348 | -356.25 | 307.83 | 1003.67 | 0.6650 |
| CD94 | 517.25 | 4252.25 | -328.5 | 862.25 | -436 | -498.25 | 728.17 | 1814.25 | 0.2404 |
| CD95 | 2270.25 | 6087.25 | 1095.5 | 889.25 | -288 | -4.25 | 1675 | 2343.02 | 0.0127 |
| CD97 | 1310.25 | 1183.25 | 132.5 | 8835.25 | -400 | -332.25 | 1788.17 | 3529.34 | 0.0452 |
| CD98 | 5648.25 | 1001.25 | 1655.5 | 6123.25 | 887 | 1140.75 | 2742.67 | 2453.34 | 0.0002 |
| CD99 | 1061.25 | 1193.25 | -224.5 | -884.75 | -171 | -366.25 | 101.33 | 834.87 | 0.9156 |
| CD99R | 261.25 | 450.25 | -248.5 | -59.75 | -292 | -300.25 | -31.5 | 318.04 | 0.6072 |
| CD100 | 298.25 | 651.25 | -154.5 | -985.75 | -471 | -535.25 | -199.5 | 596.35 | 0.3345 |
| CD102 | 1805.25 | 657.25 | -254.5 | -1073.75 | -92 | -414.25 | 104.67 | 1002.29 | 0.9262 |
| CD103 | 173.25 | 1340.25 | -69.5 | -370.75 | -466 | -415.25 | 32 | 685.86 | 0.7608 |
| CD104 | 518.25 | -238.75 | 811.5 | 242.25 | 483 | 53.75 | 311.67 | 373.14 | 0.6111 |
| CD105 | 959.25 | 953.25 | 503.5 | 6146.25 | -144 | -294.25 | 1354 | 2406.91 | 0.0478 |
| CD106 | 529.25 | -609.75 | -182.5 | 406.25 | -206 | -374.25 | -72.83 | 447.42 | 0.5317 |
| CD107a | 480.25 | 530.25 | -186.5 | 274.25 | -518 | -242.25 | 56.33 | 431.22 | 0.8041 |
| CD107b | 64.25 | 2104.25 | -41.5 | -570.75 | -438 | -418.25 | 116.67 | 1004.45 | 0.9510 |
| CD108 | 707.25 | 2929.25 | -222.5 | 2245.25 | -492 | -400.25 | 794.5 | 1468.76 | 0.1486 |
| CD109 | 72.25 | -713.75 | 28.5 | -62.75 | -588 | -535.25 | -299.83 | 349.93 | 0.1959 |
| CD112 | 558.25 | 891.25 | 215.5 | 26.25 | -559 | -349.25 | 130.5 | 545.05 | 0.9771 |
| CD114 | 189.25 | 3965.25 | -158.5 | -136.75 | -461 | -494.25 | 484 | 1723.61 | 0.4765 |
| CD116 | 166.25 | -199.75 | -249.5 | -24.75 | -316 | -335.25 | -159.83 | 194.61 | 0.3661 |
| CD117 | 445.25 | 897.25 | -244.5 | -95.75 | -393 | -401.25 | 34.67 | 525.45 | 0.7589 |
| CD118 | 382.25 | 1769.25 | -161.5 | -398.75 | -284 | -337.25 | 161.67 | 836.54 | 0.9541 |
| CD119 | 674.25 | 2819.25 | -180.5 | -635.75 | -365 | 15.75 | 388 | 1270.41 | 0.5543 |
| CD120a | 931.25 | 865.25 | -82.5 | 345.25 | -327 | -138.25 | 265.67 | 537.49 | 0.7169 |
| CD120b | -3.75 | -489.75 | 295.5 | -516.75 | -47 | -417.25 | -196.5 | 328.28 | 0.3178 |
| CD121a | 259.25 | 740.25 | -51.5 | -116.75 | -377 | -380.25 | 12.33 | 428.32 | 0.7057 |
| CD121b | 68.25 | 5621.25 | -169.5 | -24.75 | -558 | -3.25 | 822.33 | 2361.63 | 0.2477 |
| CD122 | 145.25 | -198.75 | 13.5 | 39.25 | -471 | -223.25 | -115.83 | 225.17 | 0.4406 |
| CD123 | 175.25 | 8219.25 | -67.5 | 637.25 | -497 | -358.25 | 1351.5 | 3388.61 | 0.1218 |
| CD124 | 428.25 | 905.25 | -64.5 | 405.25 | -555 | -70.25 | 174.83 | 510.06 | 0.9201 |
| CD126 | 785.25 | 836.25 | 17.5 | 4264.25 | -417 | -186.25 | 883.33 | 1733.61 | 0.1315 |
| CD127 | 399.25 | 1865.25 | -242.5 | -571.75 | -233 | -269.25 | 158 | 894.57 | 0.9626 |
| CD128b | 67.25 | -1730.75 | -36.5 | -763.75 | -306 | -94.25 | -477.33 | 681.19 | 0.0911 |
| CD130 | 1006.25 | -1025.75 | -27.5 | -306.75 | -218 | -274.25 | -141 | 657.72 | 0.4289 |
| CD132 | 404.25 | 151.25 | -47.5 | 814.25 | 108 | 263.75 | 282.33 | 301.50 | 0.6704 |
| CD134 | 37.25 | 1997.25 | -334.5 | 1005.25 | -394 | -148.25 | 360.5 | 949.77 | 0.5639 |
| CD135 | 773.25 | 999.25 | -229.5 | -896.75 | -407 | -403.25 | -27.33 | 745.26 | 0.6426 |
| CD137 | 285.25 | 359.25 | -100.5 | 144.25 | -344 | -202.25 | 23.67 | 281.86 | 0.7256 |
| CD137 ligand | 203.25 | 4010.25 | -150.5 | 1161.25 | -390 | -328.25 | 751 | 1695.31 | 0.2066 |
| CD138 | 211.25 | 4049.25 | -240.5 | -370.75 | -262 | 81.75 | 578.17 | 1714.99 | 0.3645 |
| CD140a | 915.25 | 1610.25 | -134.5 | -112.75 | -455 | 112.75 | 322.67 | 781.85 | 0.6174 |
| CD140b | 1149.25 | 7065.25 | -193.5 | -336.75 | 111 | -233.25 | 1260.33 | 2895.70 | 0.1059 |
| CD141 | 810.25 | -668.75 | 540.5 | -523.75 | -365 | -138.25 | -57.5 | 600.45 | 0.5722 |
| CD142 | 4144.25 | 1578.25 | 1977.5 | -45.75 | 156 | 1682.75 | 1582.167 | 1510.944 | 0.0033 |
| CD144 | 430.25 | -664.75 | 278.5 | 5534.25 | -279 | -89.25 | 868.33 | 2319.24 | 0.2125 |
| CD146 | 529.25 | -2607.75 | 257.5 | 383.25 | 582 | 199.75 | -109.33 | 1232.93 | 0.5464 |
| CD147 | 11858.25 | 5950.25 | 9500.5 | 1381.25 | 4543 | 8804.75 | 7006.33 | 3790.02 | 9.66E-09 |
| CD150 | 450.25 | -546.75 | -122.5 | 15807.25 | -226 | -289.25 | 2512.17 | 6521.58 | 0.0922 |
| CD151 | 2754.25 | 603.25 | 1882.5 | 82.25 | 3612 | 2834.75 | 1961.5 | 1378.25 | 0.0002 |
| CD152 | 647.25 | -84.75 | -41.5 | 5103.25 | -144 | 43.75 | 920.67 | 2069.20 | 0.1521 |
| CD153 | 286.25 | 877.25 | 53.5 | -676.75 | -391 | -153.25 | -0.67 | 545.66 | 0.6836 |
| CD154 | 918.25 | 957.25 | 184.5 | 969.25 | -312 | 290.75 | 501.33 | 530.45 | 0.3000 |
| CD158a | -80.75 | 391.25 | -124.5 | -416.75 | -228 | 758.75 | 50 | 438.51 | 0.7899 |
| CD158b | 950.25 | 7428.25 | -208.5 | 226.25 | -68 | 984.75 | 1552.17 | 2922.18 | 0.0459 |
| CD161 | 355.25 | 612.25 | 123.5 | 14.25 | -63 | -208.25 | 139 | 299.31 | 0.9966 |
| CD162 | 139.25 | 2683.25 | -0.5 | -236.75 | -512 | -238.25 | 305.83 | 1186 | 0.6848 |
| CD163 | 572.25 | 2917.25 | 225.5 | -670.75 | -274 | -191.25 | 429.83 | 1291.64 | 0.4928 |
| CD164 | 1907.25 | 3215.25 | 1080.5 | -75.75 | 1570 | 1694.75 | 1565.33 | 1075.56 | 0.0012 |
| CD165 | 909.25 | 1366.25 | 483.5 | 2510.25 | 147 | -136.25 | 880 | 961.49 | 0.0611 |
| CD166 | 1804.25 | 1783.25 | 783.5 | 835.25 | 975 | 1658.75 | 1306.67 | 490.84 | 0.0020 |
| CD171 | 184.25 | 1391.25 | 144.5 | 2491.25 | -88 | -504.25 | 603.17 | 1120.35 | 0.2523 |
| CD172b | 839.25 | 2397.25 | 112.5 | 65.25 | -215 | 35.75 | 539.17 | 976.87 | 0.3029 |
| CD177 | 198.25 | 1426.25 | -60.5 | -506.75 | -382 | -180.25 | 82.5 | 703.07 | 0.8712 |
| CD178 | 626.25 | 340.25 | -62.5 | -435.75 | -239 | 62.75 | 48.67 | 386.94 | 0.7853 |
| CD180 | 768.25 | 767.25 | 42.5 | -274.75 | -239 | -3.25 | 176.83 | 474.49 | 0.9150 |
| CD181 | 248.25 | 1342.25 | 217.5 | 1258.25 | -153 | 1905.75 | 803.17 | 809.77 | 0.0794 |
| CD183 | -345.75 | 327.25 | 110.5 | 1039.25 | 60 | -112.25 | 179.83 | 477.72 | 0.9081 |
| CD184 | 288.25 | 701.25 | -310.5 | 66.25 | -361 | -52.25 | 55.33 | 397.57 | 0.8009 |
| CD193 | 759.25 | 1103.25 | 62.5 | 1294.25 | -195 | -148.25 | 479.33 | 656.36 | 0.3419 |
| CD195 | 818.25 | 214.25 | -42.5 | 773.25 | -170 | -136.25 | 242.83 | 449.18 | 0.7633 |
| CD196 | 1514.25 | -79.75 | 373.5 | -519.75 | -170 | -221.25 | 149.5 | 728.38 | 0.9798 |
| CD197 | 152.25 | 602.25 | 529.5 | 516.25 | 629 | 38.75 | 411.33 | 250.90 | 0.4163 |
| CD200 | 6194.25 | 1567.25 | 4928.5 | 5699.25 | 1804 | 4395.75 | 4098.17 | 1969.96 | 3.49E-08 |
| CD201 | 3404.25 | 1941.25 | 2101.5 | 8521.25 | 4568 | 1507.75 | 3674 | 2628.14 | 5.61E-06 |
| CD205 | 845.25 | 59.25 | 462.5 | 409.25 | -122 | 6769.75 | 1404 | 2650.15 | 0.0539 |
| CD206 | 513.25 | 412.25 | 154.5 | 553.25 | -421 | 62.75 | 212.5 | 367.04 | 0.8300 |
| CD209 | 859.25 | 1013.25 | 281.5 | 1388.25 | -77 | 919.75 | 730.83 | 532.66 | 0.0956 |
| CD210 | -166.75 | 799.25 | 863.5 | 422.25 | -97 | -447.25 | 229 | 545.04 | 0.7978 |
| CD212 | -224.75 | -92.75 | 1337.5 | 340.25 | 83 | -513.25 | 155 | 646.52 | 0.9671 |
| CD220 | 711.25 | 241.25 | 58.5 | -601.75 | -255 | -195.25 | -6.83 | 454.42 | 0.6655 |
| CD221 | 1088.25 | 3717.25 | 640.5 | 289.25 | -313 | -137.25 | 880.83 | 1480.39 | 0.1051 |
| CD226 | -453.75 | 250.25 | 78.5 | 1054.25 | -314 | -51.25 | 94 | 535.60 | 0.8930 |
| CD227 | -328.75 | 878.25 | 824.5 | 1089.25 | -365 | 466.75 | 427.5 | 632.49 | 0.4173 |
| CD229 | 692.25 | 1112.25 | -49.5 | 863.25 | -349 | -85.25 | 364 | 599.68 | 0.5237 |
| CD231 | 885.25 | 1385.25 | -126.5 | 1469.25 | -508 | -105.25 | 500 | 853.98 | 0.3363 |
| CD235a | 368.25 | 1478.25 | -9.5 | 363.25 | -59 | -179.25 | 327 | 607.77 | 0.5948 |
| CD243 | 324.25 | 1564.25 | 263.5 | 678.25 | -270 | 456.75 | 502.83 | 607.61 | 0.3052 |
| CD244 | 710.25 | 1228.25 | 61.5 | 1150.25 | -137 | 141.75 | 525.83 | 586.48 | 0.2742 |
| CD255 | -31.75 | 679.25 | 77.5 | 994.25 | -420 | -193.25 | 184.33 | 541.63 | 0.8988 |
| CD267 | -7.75 | 2198.25 | 315.5 | 707.25 | -142 | -507.25 | 427.33 | 960.61 | 0.4543 |
| CD268 | 735.25 | 1239.25 | 394.5 | -95.75 | -334 | -118.25 | 303.5 | 602.33 | 0.6414 |
| CD271 | -443.75 | 1002.25 | 58.5 | -334.75 | -182 | -134.25 | -5.67 | 523.04 | 0.6717 |
| CD273 | 849.25 | 31.25 | 87.5 | 2690.25 | -242 | 138.75 | 592.5 | 1089.84 | 0.2590 |
| CD274 | 220.25 | 1920.25 | 371.5 | 1988.25 | -479 | 590.75 | 768.67 | 985.94 | 0.1106 |
| CD275 | -336.75 | -443.75 | 133.5 | 399.25 | -354 | 7879.75 | 1213 | 3282.53 | 0.1581 |
| CD278 | -336.75 | -15.75 | 195.5 | -585.75 | -91 | 60.75 | -128.83 | 285.43 | 0.4208 |
| CD279 | 214.25 | 989.25 | 104.5 | -136.75 | -253 | -426.25 | 82 | 501.90 | 0.8647 |
| CD282 | 863.25 | 1042.25 | -157.5 | -72.75 | -336 | -505.25 | 139 | 650.21 | 0.9968 |
| CD294 | 413.25 | -246.75 | 1109.5 | -857.75 | -88 | -350.25 | -3.33 | 682.46 | 0.6865 |
| CD304 | 1362.25 | 169.25 | -156.5 | 941.25 | 242 | -293.25 | 377.5 | 645.65 | 0.5033 |
| CD305 | 1322.25 | 338.25 | -166.5 | -732.75 | -431 | -450.25 | -20 | 750.13 | 0.6576 |
| CD309 | 1888.25 | 1499.25 | -229.5 | -531.75 | -202 | -489.25 | 322.5 | 1077.48 | 0.6447 |
| CD314 | 903.25 | 62.25 | -240.5 | 1042.25 | -364 | -374.25 | 171.5 | 641.86 | 0.9297 |
| CD321 | 1252.25 | 1626.25 | 222.5 | 4161.25 | 1298 | -242.25 | 1386.33 | 1535.60 | 0.0100 |
| CD326 | 851.25 | 3474.25 | 347.5 | 551.25 | -138 | -134.25 | 825.33 | 1354.23 | 0.1183 |
| CDw327 | 485.25 | 368.25 | -155.5 | -625.75 | -523 | -600.25 | -175.17 | 497.30 | 0.3608 |
| CDw328 | 714.25 | -9.75 | 98.5 | -543.75 | -326 | -407.25 | -79 | 458.32 | 0.5207 |
| CD329 | 530.25 | 375.25 | -329.5 | 531.25 | 1190 | 2222.75 | 753.33 | 867.69 | 0.1082 |
| CD335 | 559.25 | 64.25 | -243.5 | -990.75 | -333 | -248.25 | -198.67 | 508.62 | 0.3277 |
| CD336 | 867.25 | 106.25 | -224.5 | -395.75 | -566 | -362.25 | -95.83 | 522.86 | 0.4943 |
| CD337 | 781.25 | -193.75 | -338.5 | -539.75 | -298 | -313.25 | -150.33 | 470.13 | 0.3971 |
| CD338 | 268.25 | 1218.25 | 14.5 | -545.75 | -110 | -236.25 | 101.5 | 609.96 | 0.9115 |
| αβTCR | 436.25 | -134.75 | 98.5 | -736.75 | -515 | -552.25 | -234 | 447.80 | 0.2759 |
| γδTCR | 375.25 | 1681.25 | -176.5 | -199.75 | -581 | -294.25 | 134.17 | 819.10 | 0.9864 |
| β2-mic | 17458.25 | 5273.25 | 3261.5 | 4434.25 | 10092 | 1731.75 | 7041.83 | 5834.73 | 6.15E-06 |
| BLTR-1 | 415.25 | 57.25 | -83.5 | -379.75 | -453 | -349.25 | -132.17 | 331.44 | 0.4176 |
| CLA | -19.75 | 177.25 | 374.5 | 982.25 | 29 | -532.25 | 168.5 | 500.13 | 0.9347 |
| CLIP | 389.25 | 1146.25 | -253.5 | -28.75 | -446 | 89.75 | -132.17 | 331.44 | 0.4176 |
| CMRF-44 | 335.25 | 164.25 | -72.5 | -1091.75 | -381 | -204.25 | 149.5 | 566.28 | 0.9791 |
| CMRF-56 | -166.75 | 734.25 | 38.5 | 296.25 | -461 | -34.25 | -208.33 | 502.60 | 0.3139 |
| EGFR | 3435.25 | 2287.25 | 1397.5 | 3096.25 | 1909 | 2176.75 | 2383.67 | 756.78 | 1.26E-06 |
| fMLPR | 424.25 | 373.25 | -164.5 | -473.75 | -261 | 3956.75 | 642.5 | 1662.41 | 0.2918 |
| HPC | 110.25 | -47.75 | 20.5 | 185.25 | -222 | -351.25 | -50.83 | 203.14 | 0.5633 |
| HLA-A,B,C | 17759.25 | 5431.25 | 6765.5 | 7793.25 | 12888 | 1712.75 | 8725 | 5721.48 | 1.35E-07 |
| HLA-A2 | 17017.25 | 11454.25 | 12883.5 | 12542.25 | 129 | 4262.75 | 9714.83 | 6260.98 | 8.66E-08 |
| HLA-DQ | 649.25 | 358.25 | -7.5 | 1525.25 | -29 | -211.25 | 380.83 | 640.56 | 0.4969 |
| HLA-DR | 3785.25 | -80.75 | 210.5 | -310.75 | -209 | -354.25 | 506.83 | 1618.85 | 0.4332 |
| HLA-DR, DP, DQ | 3624.25 | 851.25 | 331.5 | 637.25 | -272 | -294.25 | 813 | 1454.00 | 0.1361 |
| iNK T | 353.25 | 206.25 | 156.5 | 267.25 | -458 | 101.75 | 104.5 | 289.08 | 0.9140 |
| Integrin β7 | 407.25 | 11638.25 | 3861.5 | 568.25 | -379 | -381.25 | 2619.17 | 4689.94 | 0.0201 |
| Disialogangli GD2 | 868.25 | 2659.25 | 417.5 | 524.25 | -91 | -276.25 | 683.67 | 1054.02 | 0.1730 |
| MIC A/B | 507.25 | 389.25 | -111.5 | 230.25 | -372 | 211.75 | 142.5 | 327.31 | 0.9950 |
| NKB1 | 295.25 | -123.75 | -146.5 | 320.25 | 107 | 1110.75 | 260.5 | 461.42 | 0.7246 |
| SSEA-1 | 108.25 | 382.25 | 213.5 | -755.75 | -508 | 8727.75 | 1361.33 | 3635.57 | 0.1403 |
| SSEA-3 | 380.25 | -850.75 | 938.5 | -323.75 | -122 | -394.25 | -62 | 632.65 | 0.5662 |
| SSEA-4 | 600.25 | 837.25 | 378.5 | -349.75 | -593 | -306.25 | 94.5 | 586.30 | 0.8952 |
| TRA-1-60 | 500.25 | -139.75 | 257.5 | -537.75 | -231 | -431.25 | -97 | 402.03 | 0.4836 |
| TRA-1-81 | 427.25 | 693.25 | -122.5 | -621.75 | -346 | -493.25 | -77.17 | 527.64 | 0.5292 |
| Vβ 8 | 1095.25 | 2333.25 | 112.5 | -907.75 | 143 | -412.25 | 394 | 1160.97 | 0.5316 |
| Vβ 28 | 413.25 | 371.25 | 394.5 | -280.75 | -209 | -508.25 | 30.17 | 409.784 | 0.7443 |

Total: 242 cell surface markers. The MFI of antibody staining including isotype control was presented after subtraction of background staining (buffer only). The difference of MFI between cell surface marker and isotype control (MFI: 140.41 ± 782.85, n = 22) was analyzed using two-tailed *t*-test. TCR: T cell receptor, β2-mic: beta-2 microglobulin (HLA-G), BLTR-1: Leukotriene B4 receptor 1, CLA: Cutaneous lymphocyte-associated antigen; CLIP: Class-II associated invariant chain peptide, EGFR: Epidermal growth factor receptor, fMLPR: fMet-Leu-Phe receptor, HPC: Hematopoietic progenitor cell surface antigen, iNK T: Invariant natural killer T cells, Disialogangli GD2: Disialoganglioside GD2, MIC A/B: MHC class I chain-related protein A and B, NKB1: A natural killer cell receptor, SSEA: Stage-specific embryonic antigen, TRA: T cell receptor alpha locus, Vβ: T cell receptor beta locus.
